# In Situ IR Spectroscopy Studies of Atomic Layer-Deposited SnO_2_ on Formamidinium-Based Lead Halide Perovskite

**DOI:** 10.1021/acsami.3c05647

**Published:** 2023-07-28

**Authors:** Andrea E. A. Bracesco, Jarvi W. P Jansen, Haibo Xue, Valerio Zardetto, Geert Brocks, Wilhelmus M. M. Kessels, Shuxia Tao, Mariadriana Creatore

**Affiliations:** † Plasma & Materials Processing, Department of Applied Physics and Science of Education, Eindhoven University of Technology (TU/e), P.O. Box 513, Eindhoven 5600 MB, Netherlands; ‡ Materials Simulation & Modelling, Department of Applied Physics and Science of Education, Eindhoven University of Technology (TU/e), P.O. Box 513, Eindhoven 5600 MB, Netherlands; § Center for Computational Energy Research, Department of Applied Physics and Science of Education, Eindhoven University of Technology (TU/e), P.O. Box 513, Eindhoven 5600 MB, Netherlands; ∥ TNO-partner in Solliance, High Tech Campus 21, Eindhoven 5656 AE, Netherlands; ⊥ Computational Materials Science, Faculty of Science and Technology and MESA+ Institute for Nanotechnology, University of Twente, P.O. Box 217, Enschede 7500 AE, Netherlands; # Eindhoven Institute of Renewable Energy Systems (EIRES), Eindhoven 5600 MB, Netherlands

**Keywords:** metal halide perovskite, atomic layer deposition, SnO_2_, infrared spectroscopy, perovskite decomposition

## Abstract

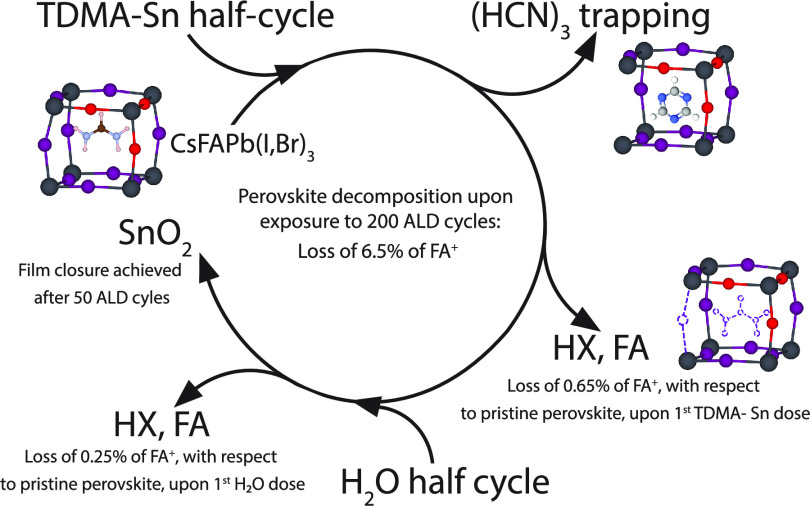

Perovskite photovoltaics has achieved conversion efficiencies of 26.0% by optimizing the optoelectronic properties of the absorber and its interfaces with charge transport layers (CTLs). However, commonly adopted organic CTLs can lead to parasitic absorption and device instability. Therefore, metal oxides like atomic layer-deposited (ALD) SnO_2_ in combination with fullerene-based electron transport layers have been introduced to enhance mechanical and thermal stability. Instead, when ALD SnO_2_ is directly processed on the absorber, i.e., without the fullerene layer, chemical modifications of the inorganic fraction of the perovskite occur, compromising the device performance. This study focuses on the organic fraction, particularly the formamidinium cation (FA^+^), in a CsFAPb(I,Br)_3_ perovskite. By employing in situ infrared spectroscopy, we investigate the impact of ALD processing on the perovskite, such as vacuum level, temperature, and exposure to half and full ALD cycles using tetrakis(dimethylamido)-Sn(IV) (TDMA-Sn) and H_2_O. We observe that exposing the absorber to vacuum conditions or water half-cycles has a negligible effect on the chemistry of the perovskite. However, prolonged exposure at 100 °C for 90 min results in a loss of 0.7% of the total formamidinium-related vibrational features compared to the pristine perovskite. Supported by density functional theory calculations, we speculate that FA^+^ deprotonates and that formamidine desorbs from the perovskite surface. Furthermore, the interaction between TDMA-Sn and FA^+^ induces more decomposition of the perovskite surface compared to vacuum, temperature, or H_2_O exposure. During the exposure to 10 ALD half-cycles of TDMA-Sn, 4% of the total FA^+^-related infrared features are lost compared to the pristine perovskite. Additionally, IR spectroscopy suggests the formation and trapping of *sym*-triazine, i.e., a decomposition product of FA^+^. These studies enable to decouple the effects occurring during direct ALD processing on the perovskite and highlight the crucial role of the Sn precursor in affecting the perovskite surface chemistry and compromising the device performance.

## 1 Introduction

Solar cells employing hybrid organic–inorganic perovskite absorbers have recently reached a photoelectric conversion efficiency of 25.7%.^1^ However, state-of-the-art perovskite-based devices always employ organic, fullerene-based charge transport layers (CTLs), such as poly[bis(4-phenyl)(2,4,6-trimethylphenyl)amine (PTAA), [6,6]-phenyl-C_61_-butyric acid methyl ester (PCBM), or (C_60_-Ih)[5,6]fullerene (C_60_), whose presence often causes optical parasitic absorption, as well as a limited mechanical and thermal stability of the devices.^2,3^ When addressing the inverted perovskite device architecture, i.e., p–i–n, thin metal oxides, such as atomic layer-deposited (ALD) SnO_2_, are processed on PCBM or C_60_ and serve as buffer layers. Specifically, they are found to impart thermal stability, prevent humidity ingress, and suppress the damage induced during the sputtering of the transparent front contact.^4−6^ ALD SnO_2_ is nowadays employed in both the p–i–n single junction and tandem perovskite/crystalline silicon, perovskite/perovskite, and perovskite/CIGS.^4,6−11^ Interestingly, there have been attempts to grow ALD SnO_2_ directly on the perovskite absorber, thereby suppressing any optical loss associated with fullerenes. So far, all literature studies reported poor solar cell performances when SnO_2_ is directly processed on the perovskite.^7−9^ The result has been attributed to the degradation of the inorganic fraction of the perovskite, accompanied by the formation of interface defects, leading either to charge recombination or to an energy barrier for electron extraction.^7−9^

All these studies investigated the same ALD process based on tetrakis(dimethylamido)-tin (TDMA-Sn) and H_2_O as the precursor and co-reactant, respectively. Hultqvist *et al.* found that the ALD processing temperature negatively affected the device performance.^8^ They reported a mass loss from a Cs_0.05_FA_0.79_MA_0.16_(I_0.83_Br_0.17_)_3_ absorber, during the pre-heating step prior to the ALD process, with a major mass loss above 90 °C. Palmstrom *et al.* reported that Cs_0.17_FA_0.83_Pb(I_0.83_Br_0.17_)_3_ suffered from the interaction with the metal–organic precursor at a temperature of 150 °C, resulting in the removal of the organic fraction of the perovskite and the formation of PbI_2_.^7^ They suggested that either a ligand exchange reaction took place between the organic fraction and the ALD precursor or that an acid–base reaction induced the deprotonation of formamidinium into formamidine. In a parallel publication, we suggested that the interaction between the halide fraction and the metal center of the ALD precursor induced the formation of PbBr_2_ and Sn^2+^ states as well as the trapping of molecular halide species.^9^ Finally, J. A. Raiford *et al.* attempted to introduce an ultrathin inorganic solution-processed PbS layer on top of the fullerene, which, however, did not prevent the decomposition of the perovskite absorber during ALD processing.^12^

This study aims to disentangle the several effects played by vacuum, processing temperature, and exposure to ALD precursor/co-reactant on the chemical stability of the perovskite, specifically on the organic cation formamidinium (FA^+^). Moreover, the investigation is complementary to a previous publication, focusing on the inorganic component of perovskite.^9^

The manuscript is organized as follows: the effect of temperature and vacuum on the chemistry of the perovskite surface is investigated and complemented by an overview of the relevant literature addressing the thermal decomposition of perovskite. FTIR and density functional theory (DFT) calculations are combined to support the attribution of specific IR absorption modes. Our results show that heat (100 °C substrate temperature) and vacuum have only a mild effect on the perovskite surface decomposition. Devices including 24 nm of ALD SnO_2_ grown at either 50 or 100 °C exhibit similar poor conversion efficiency, indicating that heat exposure is not the main parameter affecting the performance. Aided by DFT calculations, we suggest that during extended heat treatment in vacuum, the FA^+^ ions initially deprotonate into formamidine and subsequently are released from the perovskite surface in parallel with the formation of hydrogen halides. Next, we report on the exposure of perovskite to TDMA-Sn and H_2_O during half and full ALD cycles. Water exposure leads to negligible perovskite decomposition. Instead, the physi-/chemisorption of TDMA-Sn leads to the decomposition and release of deprotonated formamidinium and the formation and trapping of a byproduct, i.e., *sym*-triazine. Consecutive TDMA-Sn doses continue to modify the absorber, although with a decreasing effect due to the steric hindrance of the physisorbed molecules. Instead, if water is dosed in-between TDMA-Sn doses, the decomposition of formamidinium occurs for several ALD cycles up to SnO_2_ film closure. If the ALD process is extended above 50 cycles, only losses related to the effect of heat and vacuum are detected.

## 2 Experimental Section

### 2.1 Perovskite Absorber Synthesis

Double-side polished crystalline silicon (100) substrates with a thickness of 400 μm, a resistivity of 10–20 ohm-cm, and B doping are used. On one side of these substrates, a Cs_0.15_FA_0.85_Pb(I_0.92_Br_0.08_)_3_ perovskite is deposited. The perovskite solution is prepared by mixing a 1.33 M concentration of the following precursors: PbBr_2_ (99.9%) and PbI_2_ (99.999%), both from TCI; FAI (99.9%) and FABr (99.9%), both from Greatcell (Dyesol); and CsI (99%, Sigma-Aldrich), in anhydrous DMF:DMSO (vol ratio = 9:1), followed by stirring overnight at room temperature. The solution is then spin-coated, inside an N_2_-filled glovebox, with a two-step procedure: 10 s at 200 rpm and then 30 s at 5000 rpm. Ten seconds into the second step, 300 mL of chlorobenzene is poured on the spinning substrate. The film is then annealed on a hot plate at 100 °C for 10 min. The resulting perovskite film has a thickness of about 450 nm. For the evaluation of device performances, ALD SnO_2_ layers are deposited on top of the absorber, and the 100 nm Al electrode is thermally evaporated, using a shadow mask, at a pressure of 10^–6^ mbar.

### 2.2 Atomic Layer Deposition of SnO_2_

ALD SnO_2_ is deposited on top of the perovskite at 10^–5^ mbar in a homebuilt reactor, with specifications discussed in a previous publication.^13,14^ The metal–organic precursor used for the deposition of the SnO_2_ films is tetrakis(dimethylamido)-Sn(IV), TDMA-Sn, 99.9%, from STREM Chemicals, which is kept at 50 °C. As a co-reactant, water is used. Both the precursor and co-reactant are supplied to the ALD chamber in a vapor-drawn mode. The ALD cycle, carried out at 100 °C, consists of a 500 ms TDMA-Sn dose, followed by a purge step of 15 s, then an H_2_O vapor dose of 25 ms, followed by a purge step of 15 s. The selected dosing and purge times correspond to self-limiting conditions for the ALD process. The thickness, and refractive index, of the ALD SnO_2_ layers, grown on the c-Si substrate, is determined by means of spectroscopic ellipsometry (SE) using a Cauchy model in the region between 1.2 and 2.7 eV. The SE used is a J.A. Woollam, Inc. M2000 UV ellipsometer, and the measured growth per cycle is 0.11 (±0.01) nm.

### 2.3 Fourier Transform Infrared Spectroscopy

The FTIR measurements are performed using a Bruker Vector 22 FTIR spectrometer with a mid-infrared light source (Globar, 10,000–50 cm^–1^) and a liquid N_2_-cooled Bruker MCT 313 detector, which operates with the highest sensitivity in the spectral range between 4000 and 1000 cm^–1^. The measurements are carried out in situ in the ALD reactor during the growth process.^15,16^ KBr windows, transparent in the 4000–400 cm^–1^ range, are mounted on flanges connected to the ALD reactor chamber on both the source and detector side of the reactor. The samples are placed vertically with respect to the IR beam in the ALD reactor using a 4-axis manipulator (PREVAC). A schematic representation of the setup can be found in a previous publication and is reproduced in Figure S1a.^14^ Before each measurement, the reactor is allowed to pump down to a base pressure of 10^–5^ mbar, corresponding to a delay of 30 min before beginning the IR measurements. This step is necessary to remove residual gas and water vapor present in the reactor after the substrates have been loaded and to allow the sample to reach thermal equilibrium with the reactor chamber.

The samples, during the measurements, are exposed to vacuum and heat conditions for a maximum time of 3 h. The IR measurements are performed by averaging 4096 consecutive scans with a resolution of 8 cm^–1^, and the spectra are measured every 10 min (5 min of reactor pump-down, followed by 5 min of measurement time), as schematically shown in Figure S1b. The results are presented as the differential absorbance spectra. These are obtained by subtracting the pristine perovskite spectrum from the absorbance of the sample after the modifications, resulting in a so-called differential spectrum. For each subsequent measurement, the reference spectrum is the previous one. In this way, changes can be tracked either in time or after each precursor or co-reactant dose. In the differential spectra, negative features correspond to the removal of species from the sample surface or from the bulk since the measurements are carried out in a transmission mode and do not allow to discern between the two contributions. Positive features instead are related to the detection of new species. Baseline corrections are performed using Origin by fitting a B-spline and subtracting it from the measured absorbance to obtain a flat profile at wavenumbers where there are no vibrational modes belonging to the sample.

Additionally, integrated areas are calculated using the built-in procedure in Origin. The areas of IR bands of interest are integrated and normalized to the one corresponding to a 450 nm thick pristine perovskite.

### 2.4 DFT Calculations

DFT calculations are performed using the Vienna Ab initio Simulation Package (VASP).^17−19^ The PBE-D3(BJ) functional, combining the Perdew–Burke–Ernzerhof (PBE)^20^ exchange-correlation functional and the D3(BJ) scheme for van der Waals interactions, is used for all calculations.^21^ The outermost s, p, and d (for Pb) electrons are treated as valence electrons, whose interactions with the frozen ions’ cores are modeled within the projector-augmented wave method.

The calculations are done by using a plane wave kinetic energy cutoff of 500 eV. The energy and force convergence criteria are set to 10^–4^ eV and 0.02 eV/Å, respectively. To mimic the experimentally studied perovskite composition, Cs_0.15_FA_0.85_Pb(I_0.92_Br_0.08_)_3_, while maintaining a sensibly small number of atoms in the simulation cell, the Cs_0.125_FA_0.875_Pb(I_0.875_Br_0.125_)_3_ is selected as a model. The configurations with the most stable relative positions of Cs and FA and I and Br have been selected based on the results presented in our previous work.^22,23^ Based on this bulk compound, we construct and study the (001) surface. The latter is modeled by a repeated slab, each consisting of a 2 × 2 surface cell and 5 FAPbI_3_ units in the direction perpendicular to the surface; in this direction, the repeated slabs are separated by a vacuum spacing of 25 Å. For reference, a free-standing FA^+^ cation is also calculated. The simulation is performed in a large cubic cell with a lattice parameter of 30 Å.

**Figure 1:**
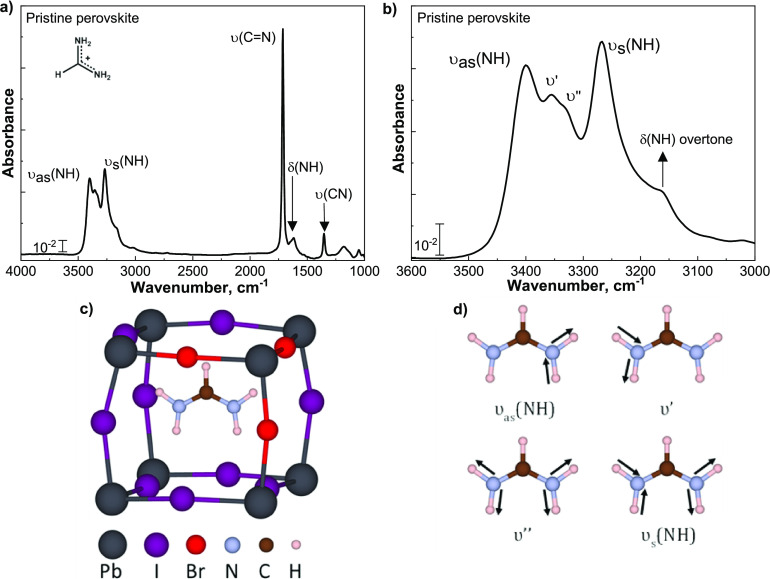
(a) IR absorbance spectrum of the pristine perovskite absorber, CsFAPb(I,Br)_3_, measured at 100 °C and 10^–5^ mbar; the assigned features correspond to the vibrational modes of FA^+^. The pristine perovskite measured at room temperature is shown in Figure S5 and presents the same absorption features. The insert shows the formamidinium chemical structure. (b) Detailed N-H stretching region between 3600 and 3000 cm^–1^. The features labeled with υ′ and υ″ are discussed in the main text. (c) Schematic illustration, based on DFT calculations, of the FA cation in a PbI/Br framework illustrating that the NH_2_ groups of FA^+^ have different chemical surroundings. (d) Illustration of the different vibrational modes of the NH_2_ groups of the FA^+^ cation.

**Figure 2:**
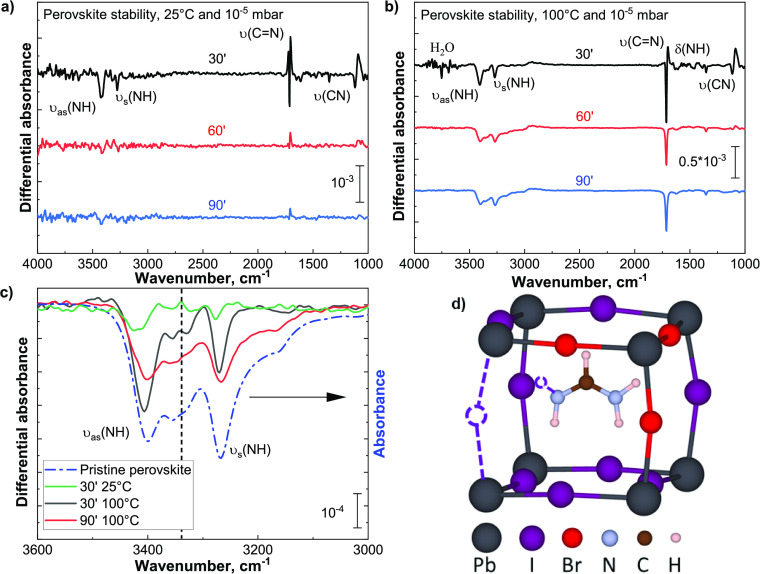
Differential absorbance spectra (with respect to the pristine perovskite spectrum shown in Figure 1a) of the 90 min exposure of perovskite to 10^–5^ mbar at (a) RT and (b) 100 °C. The duration of this exposure corresponds to a typical ALD process of 25 nm of SnO_2_. The positive/negative absorption feature at 1100 cm^–1^ corresponds to the modifications induced on the crystalline silicon (c-Si) substrate.^35^ (c) N-H stretching region for different exposure times and temperatures. To compare the results with the reference, the spectrum of the pristine perovskite has been normalized to the intensity of the 90 min exposure and mirrored with respect to the horizontal axis. The position of the two secondary stretching modes is indicated by the dotted line. Water vibrational modes can be seen above 3600 cm^–1^ and at 1600 cm^–1^. (d) Schematic illustration, based on DFT calculations, of the FA^+^ cation in a PbI/Br framework, with one HI being removed. The removed H and I atoms and the breaking bonds are represented by dashed lines.

To simulate how the perovskite is affected by the loss of a volatile species, HI, one H ion on the N atom of FA^+^, as well as one I close to that H, is removed. To compare the vibrational frequencies of the modified system to those of the pristine system, the ionic positions are kept fixed. Details of the optimized structures of the pristine and deprotonated structures are shown in the Supplementary Information and in Figures 1c,d and 2d. The vibrational modes of FA^+^ are visualized using the Jmol software.^24^

## 3 Results and Discussion

### 3.1 Pristine Perovskite Spectrum

The IR absorbance spectrum of the pristine Cs_0.15_FA_0.85_Pb(I_0.92_Br_0.08_)_3_ perovskite, measured at 100 °C and 10^–5^ mbar, is shown in Figure 1a.

The region between 3500 and 3000 cm^–1^ is characteristic of the N-H stretching vibrations of FA^+^. The two main features at 3400 and 3267 cm^–1^ correspond to the asymmetric and symmetric N-H stretching modes, υ_as_(NH) and υ_s_(NH), respectively. The υ(C=N) stretching mode of the delocalized resonating C=N bond is found at 1713 cm^–1^.^25−27^ The N-H bending mode, δ(NH), can be observed at 1619 cm^–1^. The band at 1353 cm^–1^ corresponds to the C-N stretching mode, υ(C-N).^27^

The assignment is less trivial for the remaining modes present below 1250 cm^–1^, though most certainly related to FA^+^ modes. Interestingly, no features corresponding to C-H vibrations are detected. The delocalized positive charge present in the ion enhances the dipole moment related to the N-H vibrations, masking that of C-H.^28,29^ Zooming into the N-H stretching region, Figure 1b shows a secondary peak at 3162 cm^–1^. Based on the assignment presented by Mishra *et al.* and the work from Wolff *et al*., this feature is assigned to the first overtone, 2δ(N-H), of the N-H bending mode Fermi-resonating with the N-H stretching mode.^15,30^

In addition, the secondary features, indicated by υ′ and υ″ in Figure 1b at 3355 and 3329 cm^–1^, respectively, are not explicitly assigned in the literature.^3,25−27^ To this end, we carried out DFT calculations, discussed in SI Section A. The calculations indicated that the υ′ and υ″ modes are the result of the different chemical surroundings of the two NH_2_ groups present at the two sides of the FA^+^ cation, which break the symmetry of the FA^+^ configuration. The assigned modes are summarized in Table 1 and Figure 1c,d.

**Table 1:** Summary of the Assigned Vibrational Modes of the Pristine Perovskite Film

wavenumber, cm^–1^, measured	wavenumber, cm^–1^, DFT	wavenumber, cm^–1^, literature	molecular vibration	reference
3400	3416	3410–3405	υ_as_(N-H)	(25−27,31)
3355	3378	3359 (tentatively assigned)	splitting of υ_as_(N-H)	
3329	3320		splitting of υ(N-H)	
3267	3277	3275–3270	υ_s_(N-H)	(25−27,31)
3162	3136	3171	2δ(N-H)	(26)
1713	1718	1715–1710	υ(C=N)	(25−27,31)
1619	1622	1650–1580	δ(N-H)	(27)
1353	1362	1352	υ(C-N)	(25,27)
1200–1000	1200–1000	1250–1020	FA^+^ (other modes)	(27)

### 3.2 Effect of ALD Processing Conditions

Figure 2a,b reports the chemical modifications that the perovskite absorber undergoes upon prolonged exposure to 10^–5^ mbar and room temperature or 100 °C. The latter corresponds to the standard ALD substrate temperature for the growth of SnO_2_ for PSC applications.^32−34^ As addressed in the Experimental Section, negative features correspond to either chemical modifications or loss of species from the sample. Positive features instead are related to the formation of new species at the perovskite surface. Negative features are observed across all spectra, predominantly in the N-H stretching regions and at 1713 cm^–1^. Exposure to vacuum at 25 °C leads to a negligible loss of organic species, which will be quantified later in this section, and is most certainly only surface-related, since 90 min of exposure to vacuum does not induce additional losses. On the other hand, exposure to 100 °C leads to the loss of molecular vibrational features throughout the exposure, and the damage likely extends deeper into the absorber.

Such continuous loss can be observed in Figure 2c, reporting the N-H asymmetric and symmetric stretching vibrations. In addition to the loss of the main N-H υ_s_ and υ_as_ modes during the first 30 min of the perovskite thermal treatment, two other features are present, i.e., a negative one at 1713 cm^–1^, corresponding to the loss of C=N vibrations, and a positive one at 1702 cm^–1^, which will be discussed later in the section. Prolonging the thermal stress up to 90 min leads to a spectrum resembling that of pristine perovskite and suggests a two-step degradation process. This observation, supported by the negative features of the C-N and C=N vibrations, indicates that prolonged exposure to a processing temperature of 100 °C leads to the removal of part of the FA^+^ moiety from the perovskite (sub)surface, resulting in a PbX_2_-rich phase. This is comparable to what we reported in a previous publication and by Kot *et al.* and detected through XPS measurements*.*^9,36^

As reported in Table S2 (Supporting Information), there is disagreement in the literature on the temperature onset for the decomposition of FAPI perovskite, ranging from 50 to 300 °C.^35,37−41^ Furthermore, there have been no studies on the thermal decomposition of a CsFAPb(I,Br)_3_ perovskite. Several byproducts are formed during the process, starting from the release of HX species, corresponding to the deprotonation of formamidinium into formamidine, FA, and then proceeding with the decomposition of formamidine into NH_3_, HCN or (HCN)_3_, and PbI_2_.^42−45^ We hypothesize that also in our case, during the early stages of the exposure, HX species are released, in parallel with deprotonated formamidinium, i.e., FA, primarily due to thermal stress, which causes weak intermolecular bonds to break, and vacuum, which induces the desorption of volatile species. This hypothesis is also supported by our DFT calculations, shown in SI Section B. The results indicate that upon the release of HX, Figure 2d, the secondary υ_s_(N-H) mode of the deprotonated system has a vibrational frequency overlapping that of the υ′ and υ″ modes of the pristine system. The secondary υ_as_(N-H) mode is instead absent. As a result of the overlapping contributions, the N-H stretching region of the differential spectra calculated between the pristine and deprotonated systems is characterized by a minor feature in the secondary υ_s_ and υ_as_ region. This agrees with the experimentally observed changes reported in the differential absorbance spectra in Figure 2c for the 30 min exposure to 100 °C. Furthermore, we speculate that the detected positive feature in the C=N stretching region, seen in Figure 2a,b, at 1702 cm^–1^ can be attributed to the relaxation of FA^+^ in the (PbX_6_)^4–^ cage and to changes in the surface composition. As a result, the frequency of the stretching mode of the C=N-H side of FA^+^, hydrogen-bonded with the halide fraction, is affected and its mode is detected as a positive peak.

Additionally, we quantified the above-mentioned losses during thermal treatment, as reported in Figure S8. The losses can be related to the percentage of formamidinium ions desorbed from the perovskite surface. The results show that 90 min of exposure to 100 °C in vacuum corresponds to a loss of 0.7% (±0.1%) of the vibrational modes of FA^+^ cations, as quantified with respect to the pristine perovskite spectrum. This value is more than a factor 10 higher than the 0.06% (±0.01%) lost during exposure at 25 °C.

To evaluate the effect of the substrate temperature, during the processing of SnO_2_ directly on the perovskite absorber, we deposited 24 nm of SnO_2_ at 50 °C and at 100 °C and characterized the devices. The corresponding current–voltage curves are shown in Figure S6. Both processes lead to devices exhibiting s-shaped current–voltage curves, which are indicative of non-working devices. This comparison suggests that the modifications induced by the thermal treatment most certainly play a minor role. Additionally, preliminary investigations involving compositional variations of the perovskite formulation and the use of mixed organic cations were carried out. These investigations led to results similar to those reported in this manuscript, i.e., poor device performance upon direct SnO_2_ processing on perovskite. Instead, the ALD chemistry may largely affect the perovskite (sub)surface chemistry. Therefore, in the next section, we investigate the role of H_2_O and TDMA-Sn exposure on the modification of the FA^+^ spectral features.

### 3.3 Perovskite Exposure to Precursor and Co-Reactant Doses

**Figure 3:**
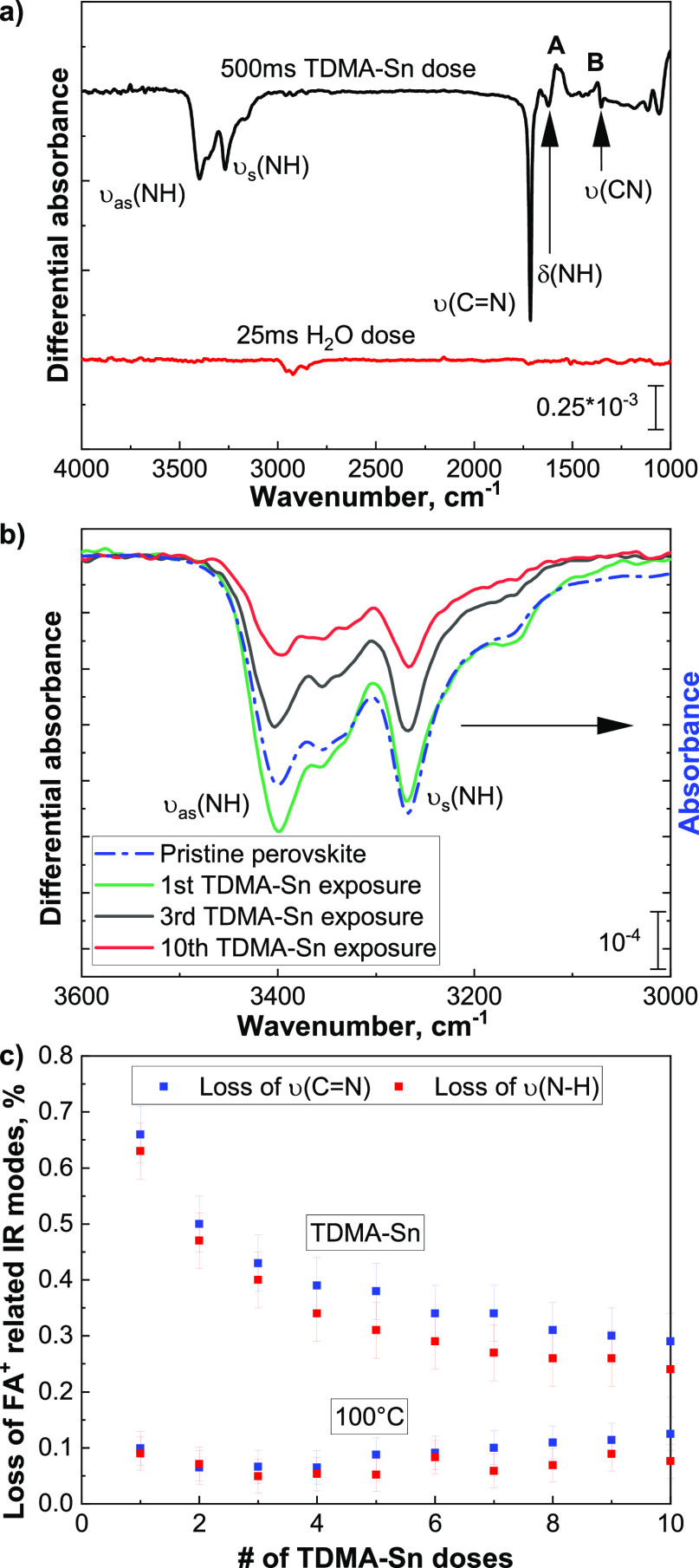
Effect of half-cycle exposure on the perovskite: (a) 25 ms of H_2_O dose and 500 ms of TDMA-Sn dose, at 100 °C and 10^–5^ mbar. The negative region visible in the 25 ms H_2_O dose spectrum, between 3000 and 2750 cm^–1^, corresponds to contaminants present on the optics of the FTIR setup and variations in the gas-phase species present in the beam path.^52^ (b) Comparison of the N-H stretching region for multiple doses of TDMA-Sn half-cycles on the perovskite film at 100 °C and 10^–5^ mbar. (c) Quantified losses, measured from the differential spectra, of the C=N stretching mode, during 10 consecutive TDMA-Sn doses and for vacuum and 100 °C.

Figure 3a shows the effect of the perovskite exposure to each ALD half-cycle, i.e., tetrakis(dimethylamido)-Sn(IV), TDMA-Sn, and H_2_O, at 100 °C. Since ALD processing on perovskite is limited in terms of processing temperature, i.e., below 100 °C, TDMA-Sn is presently the only available precursor compatible with this temperature. Other precursors such as SnCl_4_ and Sn(acac)_2_ require a much higher processing temperature, 300 and 140 °C, respectively.^46^ Furthermore, the use of co-reactants other than H_2_O, e.g., ozone or O_2_ plasma, which would enable lower processing temperatures, is detrimental to the chemical stability of perovskite.^4^ The exposure to water (Figure 3a) does not lead to any appreciable chemical modification of the perovskite absorber, also in the case of multiple H_2_O doses (Figure S10b). The stability to humidity of the adopted perovskite formulation can be attributed to the presence of Cs^+^, which reduces the size of the crystalline lattice, thereby decreasing the N-I distance and increasing the hydrogen bond strength between the formamidinium ions and the halide fraction.^47,48^ Additionally, as reported by Ning *et al.*, the partial pressure of H_2_O during the corresponding ALD half-cycle might not be sufficient to induce oxidation of the perovskite surface.^49,50^

Differently from the exposure to water doses, the perovskite exposure to the TDMA-Sn half-cycle leads to several negative features, corresponding to the υ_s_(N-H), υ_as_(N-H), υ(C=N), δ(N-H), and υ(C-N) modes. No positive features, indicative of the chemisorption of TDMA-Sn, are detected. The corresponding gas-phase IR spectrum of TDMA-Sn is reported in Figure S8, as reference. Presumably, the surface coverage of TDMA-Sn is below the sensitivity of the measurement. In the literature, it has been shown that a high surface area, such as that of a powder sample, can help to improve the detection limit. Even though the same procedure could not be adopted in our study, we still have sufficient sensitivity to evaluate the effect of the exposure of the perovskite absorber to TDMA-Sn.^51^

When we analyze the N-H stretching region in Figure 3b, the shape of the negative features resembles that of the perovskite reference, already after a single half-cycle exposure. This indicates that the formamidinium ions are decomposed due to the interaction with TDMA-Sn molecules. Furthermore, this loss slows down upon increasing the number of TDMA-Sn doses. This suggests that the initial TDMA-Sn physisorption on the perovskite surface limits its further decomposition because there are fewer available sites for further interactions. This result is supported by the losses detected via the C=N stretching mode.

In addition to the reported losses, two positive features labeled as “X” and “Y” are detected at 1548 and 1390 cm^–1^, respectively, in Figure 3a. The presence of these two positive features indicates that a byproduct of formamidine decomposition is detected at the surface of the perovskite. In Section 3.2, we presented an overview of the organic byproducts resulting from the decomposition of our perovskite formulation.^35,39,40,42−46^ HX, ammonia, and *sym*-triazine are expected to be the main species that could be released. Looking at *sym*-triazine, (HCN)_3_, its two main IR vibrational modes, i.e., ring stretch and CH bending, have been reported, for the molecule, to be in the range of 1546–1562 and 1401–1410 cm^–1^, respectively.^35,39,53−58^ These modes match with the two positive features at 1548 and 1390 cm^–1^, suggesting that the detected byproduct is (HCN)_3_. As reported by several research groups, *sym*-triazine is formed through the trimerization of formamidine in the presence of hydrogen halide species.^59−61^ We suspect that in our case, the chemisorbed TDMA-Sn prevents formamidine desorption, allowing the formation and trapping of (HCN)_3_.

Additionally, the quantified losses of the NH and C=N regions during TDMA-Sn exposure are compared to the effect of heat and vacuum (Figure 3c). The first TDMA-Sn dose leads to the loss of 0.65% (±0.05%) of the formamidinium-related signal, which is comparable to that of 90 min exposure to 100 °C and 10^–5^ mbar. After 10 consecutive TDMA-Sn half-cycles, the total amount of the FA^+^ signal lost increases to 4.0% (±0.1%). This indicates that the exposure of perovskite to TDMA-Sn molecules has a much more detrimental effect than temperature. The first dose leads to the largest relative amount of species being removed and any subsequent dose affects to a lower extent the perovskite surface, with only 0.3% for each TDMA-Sn dose after the first five half-cycle doses.

### 3.4 Perovskite Exposure to Full ALD SnO_2_ Cycles

**Figure 4:**
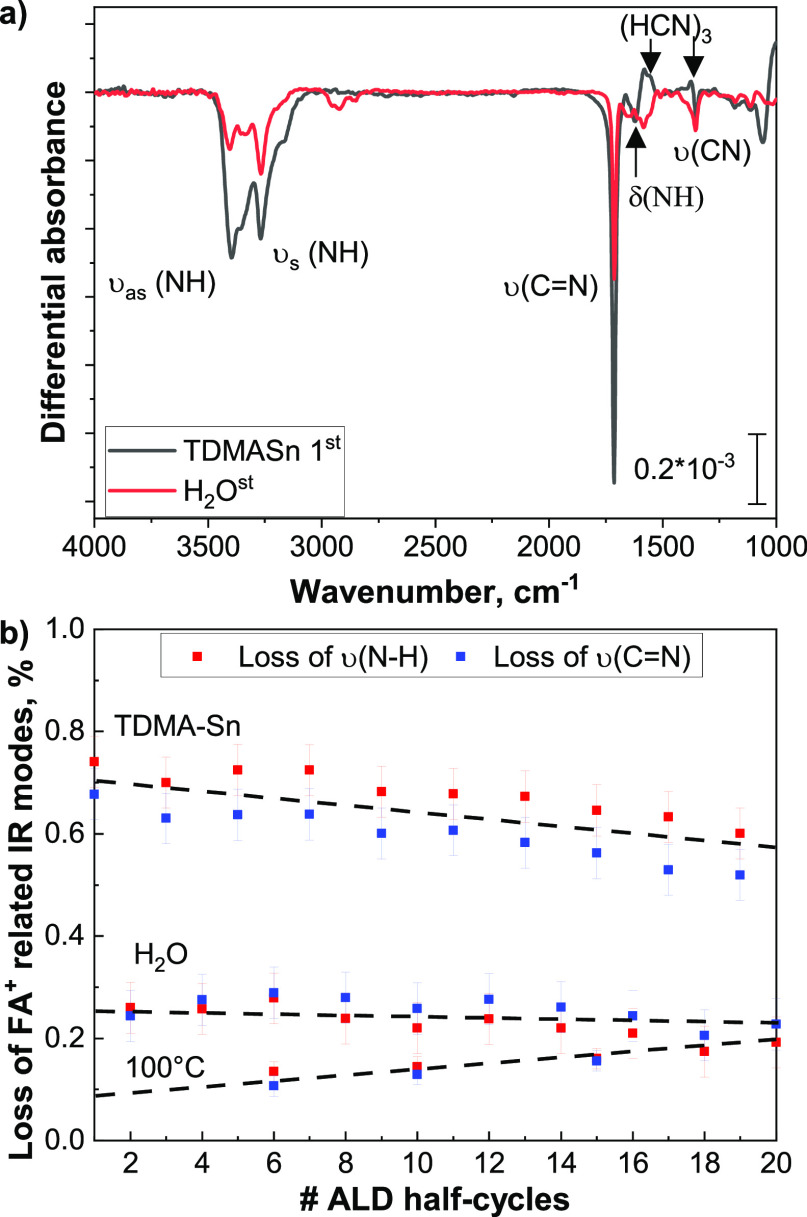
Effect of the first SnO_2_ ALD cycle on perovskite: (a) 500 ms of TDMA-Sn dose, followed by 25 ms of H_2_O dose, measured after each half-cycle at a reactor temperature of 100 °C and 10^–5^ mbar. (b) Quantified losses, measured from the differential spectra, of the C=N and N-H stretching modes for each half-cycle during 10 ALD cycles and for the prolonged exposure to 100 °C and 10^–5^ mbar.

The IR spectra corresponding to the first ALD cycle are shown in Figure 4. Those related to 10 full ALD cycles are shown in Figure S10a,b. The decomposition of FA^+^ and formation of *sym*-triazine are detected during the first ALD half-cycle (Figure 4a). Differently from the case of TDMA-Sn exposure only, the exposure to water, when dosed after the TDMA-Sn half-cycle, leads to the loss of FA^+^ cations, as the negative peaks for υ(N-H), υ(C-N), and υ(C=N) indicate.

Additionally, after the water half-cycle, negative absorption peaks are still observed at 1548 and 1380 cm^–1^. This indicates that the *sym*-triazine is either decomposed and/or desorbs from the perovskite surface during the water half-cycle. C. Grundmann *et al.* reported that if a 10% solution of *sym*-triazine in H_2_O is kept at room temperature, it decomposes through an autocatalytic reaction, thanks to its sensitivity to nucleophilic attack.^60^ However, in our case, it is more likely that the trapped molecules would desorb from the perovskite surface, due to the low boiling point.^60^ Additionally, during the early stages of SnO_2_ growth, the layer does not fully cover the surface of the perovskite, thus allowing for the desorption of volatile species. Furthermore, as reported earlier, during multiple consecutive TDMA-Sn half-cycles, no additional positive features could be detected in the FTIR spectra after the first dose. This indicates, as discussed in the previous section, that the chemisorbed TDMA-Sn molecules occupy the available sites and form a “barrier” that limits additional interactions with subsequently dosed precursor molecules. Instead, if water is dosed between TDMA-Sn half-cycles, the steric hindrance offered by TDMA-Sn, with its initial physisorption, is lost due to the removal of the organic ligands. As a result, the subsequent TDMA-Sn doses will be able to reach the perovskite surface and interact with it, as shown in Figure S11b, thereby leading to more damage than previously reported for the multiple TDMA-Sn doses alone. Once film closure occurs, the TDMA-Sn molecules will cease interacting with the perovskite surface and only the effect of exposure to heat should be detected.

Additionally, Figure 4b shows the quantified losses during the first 10 ALD cycles. As can be seen, the trends are different from those shown in Figure 3b. During multiple consecutive TDMA-Sn half-cycles, the exponential decay of the FA^+^ removal was observed. During a full-cycle fashion, a linearly decreasing trend is observed. The FA^+^ losses during the exposure to TDMA-Sn doses decrease from 0.7 to 0.6% within 10 cycles, suggesting that the SnO_2_ layer (with an estimated thickness of 1.2 nm) does not yet fully cover the perovskite surface and thus enables further interaction of TDMA-Sn with FA^+^. Additionally, the water half-cycle trend shows that FA^+^ losses have a constant trend with about 0.3% FA^+^ removed during each half-cycle, indicating that, differently from a pristine perovskite, the decomposed perovskite surface is more prone to oxidation.

### 3.5 Effect of the Prolonged SnO_2_ ALD Process

**Figure 5:**
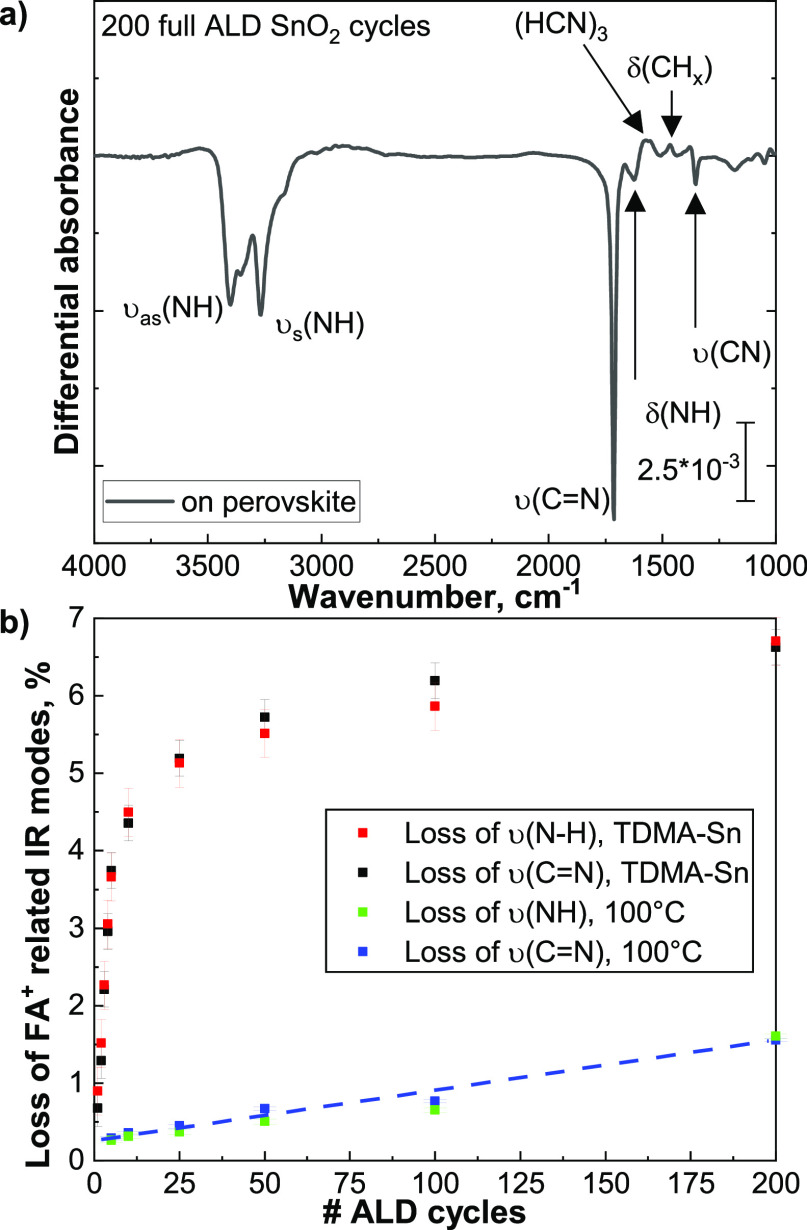
(a) FTIR spectra of 200 ALD cycles of SnO_2_, corresponding to 24 nm, grown directly on the perovskite absorber and on a c-Si substrate. The vibrational modes belonging to the removal of FA^+^ (negative peaks), the inclusion of methyl groups from TDMA-Sn ligands, and the trapped *sym*-triazine (positive peaks) are assigned. (b) Quantified losses of the C=N and N-H stretching modes calculated for multiple SnO_2_ ALD cycles and for the prolonged exposure to 100 °C and 10^–5^ mbar.

Figure 5a shows the combined effect that 200 ALD cycles, corresponding to a film thickness of 24 nm of SnO_2_ as grown on c-Si, have on the perovskite absorber. The IR spectrum is characterized by negative absorption features related to the decomposition and desorption of FA^+^ species from the perovskite surface. Moreover, positive features assigned to *sym*-triazine are still visible, indicating that a fraction of the formed molecules is trapped at the perovskite/SnO_2_ interface. This result shows that the water half-cycle only partially decomposes or allows for the desorption of the byproducts.

In addition to these modifications, two other features were detected. First, the incorporation of TDMA-Sn ligands is confirmed by the detection of vibrational modes at 1466 cm^–1^, corresponding to the bending mode, δ(CH), of the CH_*X*_ groups. XPS reveals the presence of C and N impurities in the film, at 6 and 2 at. %, respectively. Such incorporation was also previously reported by Mackus *et al.*(51) Also, we observe the presence of hydroxyl groups, resulting from the surface hydroxylation of the metal oxide layer after abstraction of the residual TDMA-Sn ligands when the surface is exposed to the water half-cycle. The corresponding vibrational modes are found in the 3600–3500 cm^–1^ region where O-H vibrations are detected, as shown in Figure S12.

Finally, Figure 5b reports the losses of formamidinium IR modes during the ALD process and compares it with the effect of exposure to temperature and vacuum. During the first 25 cycles, there is a sharp increase in the loss of FA^+^, up to 5%. In the following cycles, up to 50, the relative amount of species removed during each cycle decreases. In total, the growth of 24 nm of SnO_2_ induces the loss of 6.5% (±0.5%) of the total amount of formamidinium ions present in the 450 nm thick perovskite layer. This is about four times larger than the amount of species lost during the exposure to temperature and vacuum, which is 1.6% (±0.1). Comparing the quantified losses related to heat and vacuum exposure with that of full ALD cycles allows to evaluate whether the absorber is affected after the surface has been fully covered by a SnO_2_ layer. The slopes for the ALD cycles and for the exposure to heat and vacuum are 0.008 (±0.0016) for the N-H stretching region and 0.006 (±0.0015) for the C=N region. This comparison indicates that the detected losses after the SnO_2_ has fully covered the perovskite surface are related to the continuous thermal stress that the perovskite is subjected to and to the decomposition of the organic fraction taking place underneath the perovskite/SnO_2_ interface. Table 2 provides a summary of the findings of the decomposition of the perovskite absorber upon ALD processing.

**Table 2:** Effect(s) of Exposure to ALD Processing Conditions, Precursor, Co-Reactant, and ALD Cycles on the Perovskite Absorber

exposure to	effect on the perovskite absorber	additional details
100 °C, 10^–5^ mbar (Figure 2b)	• FA^+^ deprotonation into FA, accompanied by the release of HX species: 0.7% of FA^+^ moiety lost upon 90 min of exposure, 1.6% of FA^+^ moiety lost upon 180 min of exposure	
H_2_O pulses (Figure 3a and Figure S10b)	• No losses detected within IR detection limit	
TDMA-Sn pulses (Figure 3a–c and Figure S10a)	• FA^+^ decomposition and abstraction: 0.65% of FA^+^ moiety lost during 1 pulse, 4% of FA^+^ moiety lost after 10 pulses• Formation and trapping of *sym*-triazine	Adsorbed TDMA-Sn molecules form a “barrier” preventing continuous perovskite decomposition with subsequent precursor doses.
1 ALD cycle (Figure 4a,b)	• FA^+^ decomposition and abstraction: 0.65% of FA^+^ loss upon 1 TDMA-Sn half-cycle, 0.25% of FA^+^ loss upon H_2_O half-cycle• *sym*-triazine desorption	H_2_O removes the barrier effect provided by the adsorbed TDMA-Sn molecules.
<200 ALD cycles (Figure 5a,b and Figure S11a–c)	• FA^+^ decomposition and abstraction: 5% of FA^+^ moiety lost upon 10 ALD cycles, 6.5% of FA^+^ moiety lost upon 200 cycles• *sym*-triazine trapping at the SnO_2_/perovskite interface	Full surface coverage is achieved after 50 ALD cycles.

## 4 Conclusions

In summary, the chemical modifications occurring to the surface of a perovskite absorber during ALD SnO_2_ processing were investigated by means of in situ infrared spectroscopy. The effects of the different processing parameters such as substrate temperature and vacuum level, as well as half and full ALD SnO_2_ cycles, on the organic fraction of the perovskite were unraveled.

The exposure of the perovskite surface to water vapor, i.e., an ALD half-cycle, negligibly affects the organic fraction of the perovskite. Instead, with the support of DFT calculations, we speculate that the perovskite absorber exposure to vacuum, 10^–5^ mbar, and a temperature of 100 °C induces the deprotonation of formamidinium, toward formamidine along with the release of HX species, with X being the neighboring halide atom, hydrogen-bonded to the formamidinium cation. Additionally, if the exposure is prolonged, the whole formamidine is abstracted from the perovskite surface. Within 90 min of thermal stress in vacuum, up to 0.7% (±0.1%) of the total organic fraction moiety, as measured on the pristine perovskite absorber, is lost from the perovskite (sub)surface. Nevertheless, heat and vacuum are not deemed to be the ALD parameters compromising the performance of PSCs, as indicated by the current–voltage characteristics of devices employing ALD SnO_2_ ETLs grown at either 50 or 100 °C.

Instead, the ALD TDMA-Sn precursor leads to extensive formamidinium decomposition, with a loss equivalent to 0.65% (±0.05%) of the organic moiety of the perovskite absorber within one TDMA-Sn dose. If a full ALD cycle is carried out, the losses increase and within the first 25 cycles, 5% of the FA^+^-related feature is abstracted from the perovskite (sub)surface. Furthermore, during the perovskite/TDMA-Sn interaction, the FA^+^ cations are decomposed, leading to the formation, and trapping, of *sym*-triazine.

The insights gained throughout this study are useful to evaluate how the chemical interaction between the SnO_2_ ALD precursor and the perovskite organic fraction is responsible for surface decomposition and ultimately for non-working devices. These results allow to disentangle the several effects played by vacuum, processing temperature, and perovskite exposure to ALD precursor/co-reactant and lead to the conclusion that the direct interaction between the Sn-ALD precursor and the perovskite organic fraction should be avoided to preserve the integrity of the perovskite (sub)surface.

## Supporting Information Available

Details related to the DFT calculations for the pristine perovskite vibrational modes and for the deprotonated system; schematic diagram of the ALD reactor with the highlighted in situ IR spectroscopic capabilities and the ALD-FTIR procedure used in the experimental investigation; FTIR absorption spectra of the pristine perovskite measured at room temperature and 100 °C, the c-Si reference spectrum, the gas-phase TDMA-Sn spectra measured at room temperature and 100 °C, *J*–*V* curves of perovskite solar cells employing 24 nm of ALD SnO_2_ processed directly on top of the absorber, the trend of the calculated FA^+^-related losses during extended exposure to 100 °C and 10^–5^ mbar, the differential absorbance spectra of 10 consecutive TDMA-Sn doses, 10 consecutive H_2_O doses, 10 consecutive ALD SnO_2_ cycles measured every half-cycle on perovskite and 5 consecutive ALD SnO2 cycles on c-Si, and 200 ALD SnO_2_ cycles on perovskite (PDF)
