# Supplementary material for: In Situ IR Spectroscopy Studies of Atomic Layer-Deposited SnO2 on Formamidinium-Based Lead Halide Perovskite
Source: ACS Appl Mater Interfaces. 2023 Jul 28;15(31):38018–28. doi: 10.1021/acsami.3c05647 (PMC10416150; doi:10.1021/acsami.3c05647)
Supplement: Supplementary file 1 — am3c05647_si_001.pdf [file am3c05647_si_001.pdf]

Supporting Information

In-situ IR spectroscopy studies of atomic layer deposited SnO_2 on formamidinium-based lead halide perovskite

Andrea E.A. Bracesco[1], Jarvi W.P Jansen[1], Haibo Xue[2,3], Valerio Zardetto[4], Geert Brocks[2,3,5], Wilhelmus M.M. Kessels[1,6], Shuxia Tao[2,3], Mariadriana Creatore[1,6]*

[1] Plasma & Materials Processing, Department of Applied Physics and Science of Education, Eindhoven University of Technology (TU/e), P.O. Box 513, 5600 MB Eindhoven, The Netherlands

[2] Materials Simulation & Modelling, Department of Applied Physics and Science of Education, Eindhoven University of Technology (TU/e), P.O. Box 513, 5600 MB Eindhoven, The Netherlands

[3] Center for Computational Energy Research, Department of Applied Physics and Science of Education, Eindhoven University of Technology (TU/e), P.O. Box 513, 5600 MB Eindhoven, The Netherlands

[4] TNO-partner in Solliance, High Tech Campus 21, 5656 AE Eindhoven, The Netherlands

[5] Computational Materials Science, Faculty of Science and Technology and MESA+ Institute for Nanotechnology, University of Twente, P.O. Box 217, 7500 AE Enschede, The Netherlands

[6] Eindhoven Institute of Renewable Energy Systems (EIRES), 5600 MB Eindhoven, The Netherlands,

m.creatore@tue.nl

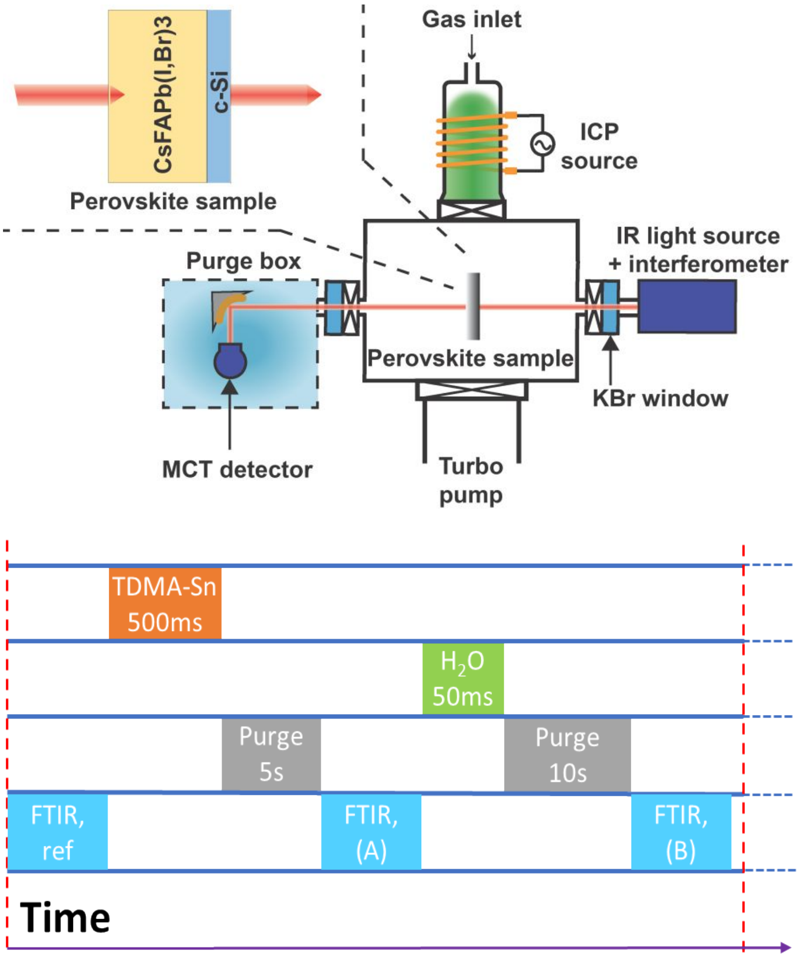

Figure S1. *Schematic illustration of the home-built ALD reactor used for the in-situ transmission IR spectroscopy measurements. The different components of the system are highlighted and include IR source, MCT detector, ICP plasma source, and turbo-pump. Additional details can be found in a previous publication.*[1] *During our experimental investigation, the perovskite absorber is processed on top of a c-Si substrate. The ALD cycle used during the experimental investigation is shown schematically. The FTIR differential spectra are reported by subtracting the spectrum of the perovskite from measurement FTIR (A) or FTIR (B).*[2] *The first spectrum measured during each experiment is taken as reference.*

A. Assignment of (Cs,FA)Pb(I,Br)$_3$ IR vibrational modes aided by DFT calculations

Density functional theory (DFT) calculations were performed to confirm the attribution of the infrared modes of FA$^+$, which are highlighted in the spectrum shown in Figure 1b. The optimized slab model of the perovskite structure is shown in Figure SI 2a. The calculated vibrational modes with their corresponding wavenumbers are summarized in Table S1. For reference, a free-standing formamidinium cation was also

calculated and taken as the simplest model for the assignment of the vibrational modes of FA^+ in the perovskite structure.

Based on the results, it was determined that there are four different N-H stretching modes belonging to the four H bonded to the two N in each FA^+. Each of the two H atoms bonded to the same N can form one symmetric and one asymmetric combination, splitting the vibrations of each side of the FA^+ cation into asymmetric, υ_{as}(N-H), and symmetric, υ_{s}(N-H), vibrational modes, as illustrated in Figure 1d. For the free-standing FA^+ cation, considered thus without any influence from the perovskite surroundings, the splitting within each stretching mode is small, with 5 cm^{-1} and 17 cm^{-1} difference for υ_{as}(N-H) and υ_{s}(N-H), respectively, as shown in Table S1. This indicates that each of these two modes is nearly doubly degenerate, owing to the symmetric configuration of the FA^+ cation. In the mixed compound $(CS,FA)Pb(I,Br)_3$, this symmetry is broken due to the different chemical surroundings of the two NH_2 groups present at the two sides of the FA^+ cation, as illustrated in Figure 1c. As a result, the splitting within the asymmetric and symmetric modes is increased to 38 cm^{-1} and 43 cm^{-1}, respectively. As a result, this leads to four different vibrational modes, υ_{as}(N-H), υ', υ'', and υ_{s}(N-H) are detected, as shown in Figure 1d.

Table S1. *Vibrational modes and wavenumbers (cm^{-1}) of a free-standing FA^+ cation and of a FA^+ cation in the surface layer of the $(FA,Cs)Pb(I,Br)_3$ perovskite.*

**Structure**	**υ_{as}(N-H)**		**υ_{s}(N-H)**		**2δ(N-H)**	**υ(C=N)**
Free-standing FA^+	3530	3525	3417	3400	3104	1771
$(Cs,FA)Pb(I,Br)_3$	3416	3378	3320	3277	3136	1718
**Structure**	**δ(N-H)**	**υ(N-H)**	**υ(C-N)**		**FA^+ other modes**	
Free-standing FA^+	1690	1617	1402	1390	1159	1085
$(Cs,FA)Pb(I,Br)_3$	1622	1567	1362	1351	1127	1051

These results are comparable to the assignment given by Hills-Kimball *et al.,* where they suggested that the N-H stretching vibrations are affected by different hydrogen-bond strengths between the H in the FA^+ and halides present in the inorganic perovskite cage.[3–5] The above analysis well explains the secondary features, υ' and υ '', measured by the experiment, which has a 45 cm^{-1} and 62 cm^{-1} difference with respect to the υ_{as}(N-H) and υ_{s}(N-H), respectively, as shown in Table I of the main text. In addition to the N-H stretching frequencies, also the calculated values for the mixed halide perovskite $(Cs,FA)Pb(I,Br)_3$ are generally in good accordance with those experimentally measured.

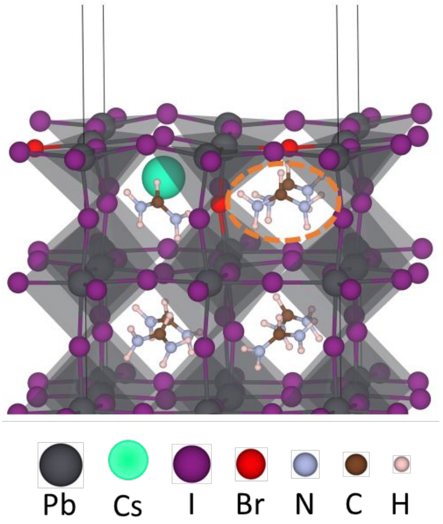

Figure S2. *Optimized structures of a slab of $Cs_{0.05}FA_{0.95}Pb(I_{0.95}Br_{0.05})_3$. The FA^+ cation, whose vibrational frequencies are studied, is highlighted by an orange dashed circle.*

B. Deprotonation of FA^+ and supporting results from DFT simulations

As reported in Table S2, the release of HX species is independent of the temperature at which the perovskite, or its simpler constituent FAI, is exposed and begins from the deprotonation of formamidinium into formamidine. As can be seen, the reported temperature onsets for the release of decomposition byproducts, vary widely depending on the study and oscillate between 50 and 360 °C. [7-10] This process is caused by the intrinsic high reactivity of the delocalized double bond.[11] The resulting formamidine molecules, having lost one of their hydrogen bonds, are more likely to be abstracted from the perovskite surface which becomes organic-deficient.[12] To precisely compare the vibration frequencies of the deprotonated system to those of the pristine system, the optimized structure of the deprotonated system, with one Hi or HBr removed, is calculated and shown in Figure S3.

Table S2. *Literature-reported thermal decomposition onset and released byproducts of FA^+-based perovskites.*

Perovskite	Onset decomposition Temperature, °C	Decomposition product(s)	Reference
FAI	280	$(HCN)_3$, NH_4I	7
	300	HCN, NH_3, HI	7
FAI	>50	$(HCN)_3$, HCN, HI	10
$FAPbX_3$	60	HCN, NH_3, HX	9
	>95	$(HCN)_3$, NH_3, HX	9
$FAPbI_3$	300-330	HI, $(HCN)_3$, NH_3	8
	360	HI, HCN, NH_3	8

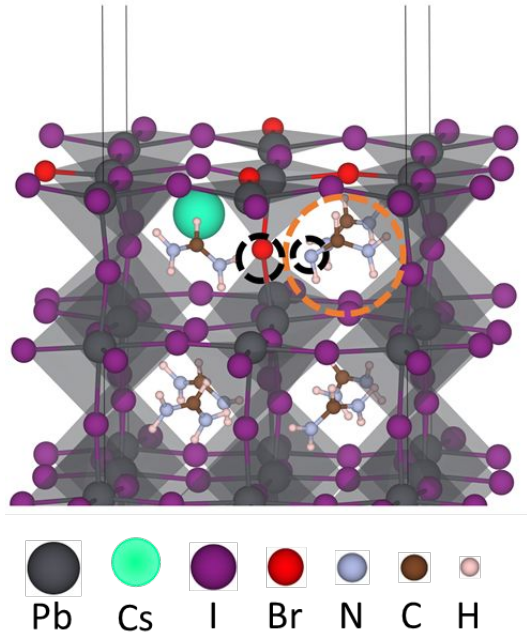

Figure S3. *Optimized slab of* $Cs_{0.05}FA_{0.95}Pb(I_{0.95}Br_{0.05})_3$ *with one HI removed. The studied* FA^+ *cation is highlighted by an orange dashed circle. The removed H and I atoms are indicated by the black dashed circles.*

The comparison between the vibrational modes of the deprotonated FA and the pristine FA^+ cation is given in Table S3. Noticeably, three vibrational modes are absent in the deprotonated FA system at 3378, 1718 and 1127 cm^{-1}. Assuming that a fraction of the formamidinium moiety is converted to formamidine due to the deprotonation, it would be expected to have negative features arise in correspondence with each of the vibrational modes of FA^+ and positive ones corresponding to FA. Additionally, as the DFT calculations show, some of the vibrational modes in the FA system appear to be shifted (and even overlap) with those of the pristine model.

Comparing the negative features of the experimentally measured pristine perovskite spectrum with that of the heat-treated one matches this behavior. Specifically, looking at the secondary features of υ_{as}(N-H) and υ_s(N-H) at 3355 and 3329 cm^{-1}, respectively, we do not detect them as negative features. This is due to, as shown by the DFT calculations, the fact that the secondary υ_s(N-H) mode of the deprotonated system vibrates with a frequency in between the two pristine ones, thus overlapping with their negative features and resulting in a smaller contribution to these losses in the IR spectrum, similar to what we detected experimentally. These results support the hypothesis of formamidinium undergoing deprotonation, coupled with the release of HI, during ALD processing. Additionally, as shown in Figure S4, the deprotonation of the FA^+ cation results in the pinning of the C=N bond on the side that loses the hydrogen atom. In the IR spectrum, such change would be shown as a negative peak corresponding to the loss of the delocalized resonating C=N bond, as also determined through the DFT calculations reported above. It also would give rise to a positive peak caused by shifts in the vibrational frequencies of the remaining C=N bonds, affected by the atomic ordering and subsequent changes in the surface composition. For the remainder of the vibrational modes, their intensity is much lower than those discussed above, and their evaluation is less trivial.

Table S3. *Comparison of the IR modes of* FA^+ *in the pristine perovskite and after HI removal. For comparison, the experimentally measured values of the pristine perovskite and the one after heat-exposure are also provided.*

**Perovskite**	**υ_{as}(N-H)**		**υ_s(N-H)**		**2δ(N-H)**	**υ(C=N)**
Pristine (calc.)	3416	3378	3320	3277	3136	1718
HI removed (calc.)	3441	*absent*	3368	3287	3110	*absent*
Pristine (exp.)	3400	3355	3329	3267	3162	1713
Heat-exposed (exp.)	*negative feature*	/	/	*negative feature*	/	*negative feature*
**Perovskite**	**δ(N-H)**	**υ(N-H)**	**υ(C-N)**		**fingerprint**	

Pristine (calc.)	1622	1567	1362	1351	1127	1051
HI removed (calc.)	1627	1538	1334	1304	*absent*	1070
Pristine (exp.)	1619	/	1353	/	/	/
Heat-exposed (exp.)	*negative feature*	/	*negative feature*	/	/	/

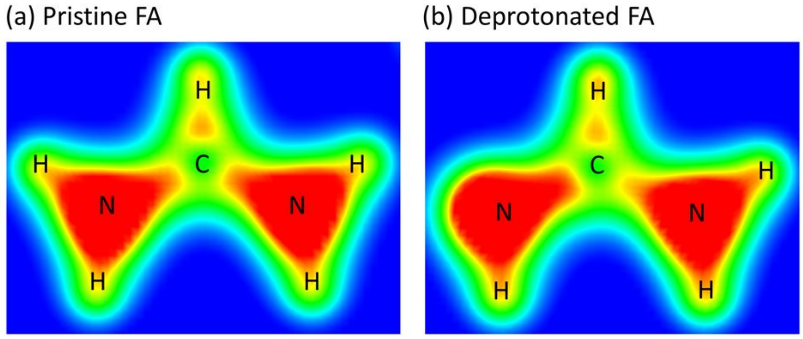

Figure S4. *Charge density of the pristine (a) and deprotonated (b) FA, where the red color indicates higher density of charge distribution. It shows that the charge distribution on pristine FA is delocalized between two C-N bonds, while it is slightly more localized on the side of the C=N bond in the deprotonated FA.*

C. Additional figures

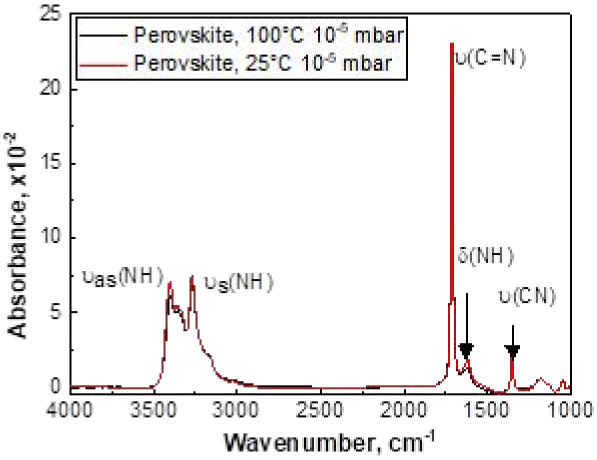

Figure S5. *FTIR absorption spectra of the pristine perovskite sample measured in two different conditions: at room temperature at 10^{-5} mbar and at 100C at 10^{-5} mbar, with the latter being the conditions used for the ALD process for the growth of SnO_2.*

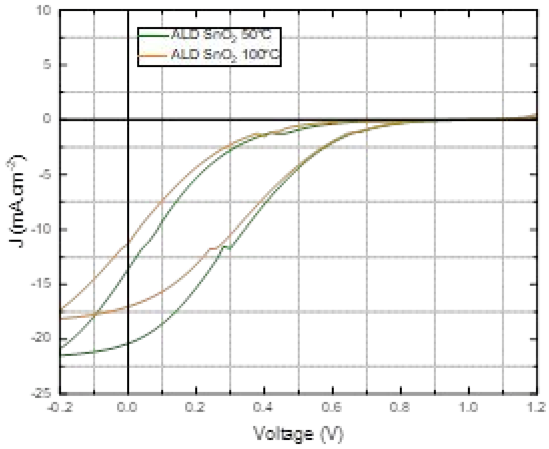

Figure S6. *JV curves of PSCs employing SnO_2 directly processed on the perovskite absorber.*

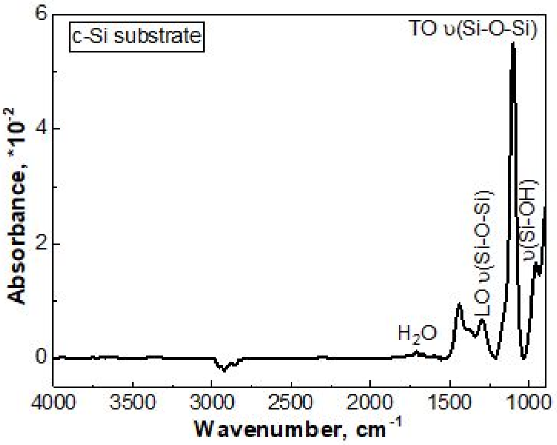

Figure S7. *Measured FTIR absorbance of a pristine c-Si substrate at 100°C at 10^{-5} mbar, with the latter being the conditions used for the ALD process for the growth of SnO_2. An intense absorption peak is observed at 1103 cm^{-1} which is assigned to the Si-O-Si asymmetric stretching. Additionally, secondary vibrations are present in the shoulder of this peak, like Si-OH or strained Si-O-Si bonds.*

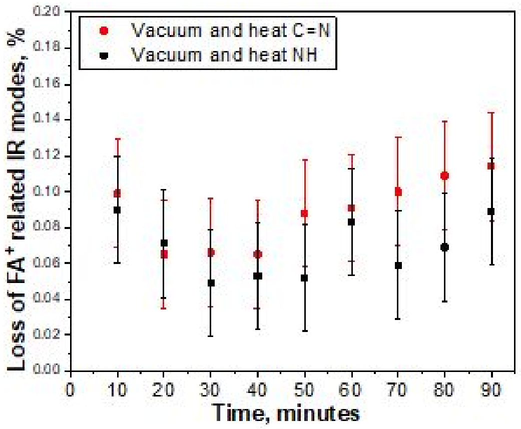

Figure S8. *Trends of the calculated amount, in percentage, of FA^+ species lost during the extended exposure to 10^{-5} mbar and 100°C.*

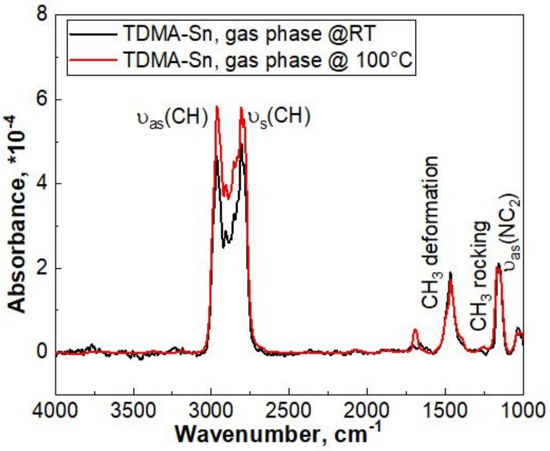

Figure S9. *TDMA-Sn molecules gas phase FTIR absorbance spectra measured in two different conditions: at room temperature at 10^{-5} mbar and at 100°C at 10^{-5} mbar, with the latter being the conditions used for the ALD process for the growth of SnO_2.*

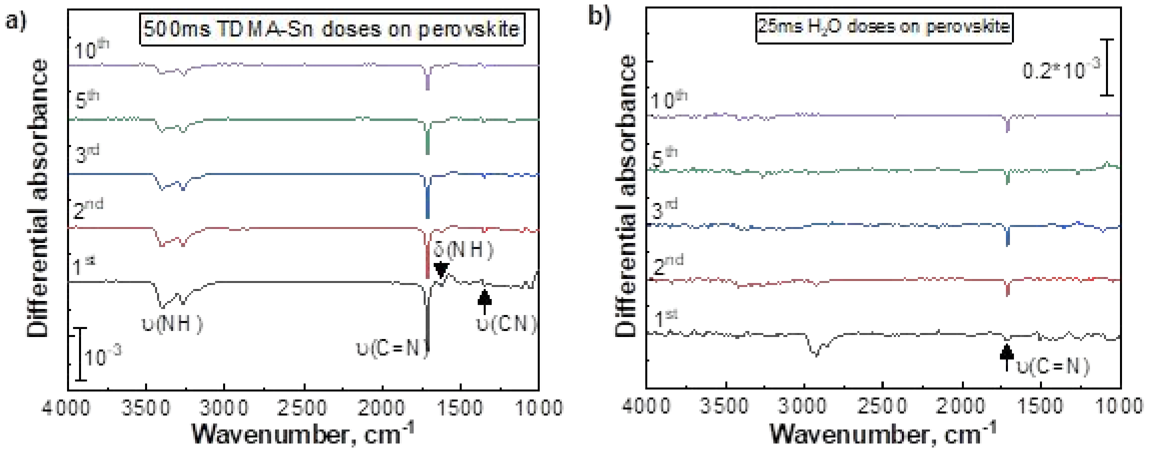

Figure S10. *a) 10 consecutive TDMA-Sn dosing steps on top of the perovskite absorber. b) 10 consecutive H_2O dosing steps on top of the perovskite absorber.*

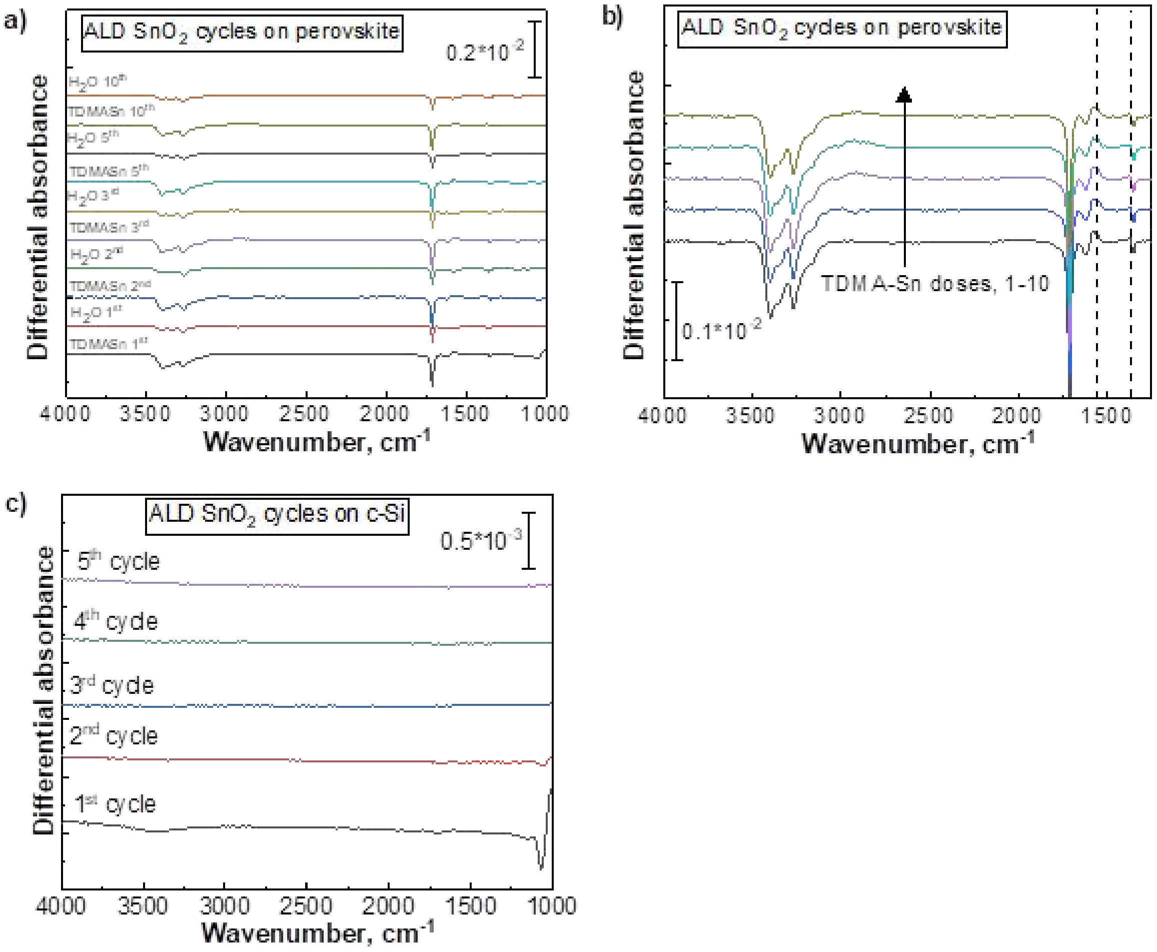

Figure S11. *a) 10 consecutive ALD SnO_2 full cycles processed on top of the perovskite absorber, measured every half-cycle. b) 10 consecutive ALD SnO_2 full cycles, shown only the TDMA-Sn doses and indicated, by the dotted lines, the position of the two positive features corresponding to the formation of sym-triazine. c) 5 consecutive ALD SnO_2 full cycles processed on top of the c-Si substrate, measure after every H_2O half-cycle.*

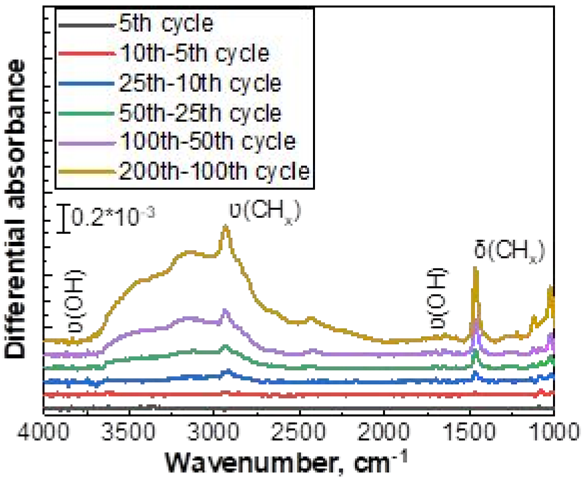

Figure S12. *200 ALD SnO_2 cycles on a c-Si substrate.*

List of references.

1. Merkx, M.J.M.; Sandoval, T.E.; Hausmann, D.M.; Kessels, W.M.M.; Mackus, A.J.M.; Mechanism of Precursor Blocking by Acetylacetone Inhibitor Molecules during Area-Selective Atomic Layer Deposition of SiO_2. *Chemistry of Materials.* 2020, 32(8), 3335-3345. doi:10.1021/acs.chemmater.9b02992
2. Koushik, D.; Hazendonk, L.; Zardetto, V.; Vandalon, V.; Verheijen, M.A.; Kessels, W.M.M.; Creatore, M.; Chemical Analysis of the Interface between Hybrid Organic-Inorganic Perovskite and Atomic Layer Deposited Al_2O_3. *ACS Appl. Mater. Interfaces.* 2019, 11(5), 5526-5535. doi:10.1021/acsami.8b18307
3. Solanki, A.; Tavakoli, M.M.; Xu, Q.; Dintakurti, S.S.H.; Lim, S.S.; Bagui, A.; Hanna, J.V.; Kong, J.; Sum, T.C.; Heavy Water Additive in Formamidinium: A Novel Approach to Enhance Perovskite Solar Cell Efficiency. *Advanced Materials.* 2020, 32(23). doi:10.1002/adma.201907864
4. Hills-Kimball, K.; Nagaoka, Y.; Cao, C.; Chaykovsky, E.; Chen, O.; Synthesis of formamidinium lead halide perovskite nanocrystals through solid-liquid-solid cation exchange. *J. Mater. Chem. C* 2017, 5(23), 5680-5684. doi:10.1039/c7tc00598a
5. Wang, P.; Guan, J.; Galeschuk, D.T.K.; Yao, Y.; He, C.F.; Jiang, S.; Zhang, S.; Liu, Y.; Jin, M.; Jin, C.; Song, Y.; Pressure Induced Polymorphic, Optical and Electronic Transitions of Formamidinium Lead Iodide Perovskite. *J. Phys. Chem. Lett.* 2017, 8, 10, 2119-2125
6. Li, N.; Tao, S.; Chen, Y.; Niu, X.; Onwudinanti, C.K.; Hu, C.; Cation and anion immobilization through chemical bonding enhancement with fluorides for stable halide perovskite solar cells. *Nat. Energy.* 2019, 4(5), 408-415. doi:10.1038/s41560-019-0382-6
7. Thampy, S.; Zhang, B.; Park, J.G.; Hong, K.H.; Hsu, J.W.P.; Bulk and interfacial decomposition of formamidinium iodide ($HC(NH_2)_2I$) in contact with metal oxide. *Mater Adv.* 2020, 1(9), 3349-3357. doi:10.1039/d0ma00624f
8. Ma, L.; Guo, D.; Li, M.; Wang, C.; Zhou, Z.; Zhang, F.; Ao, Z.; Nie, Z.; Temperature-dependent thermal decomposition pathway of organic-inorganic halide perovskite materials. *Chemistry of Materials.* 2019, 31(20), 8515-8522. doi:10.1021/acs.chemmater.9b03190
9. Juarez-Perez, E.J.; Ono, L.K.; Qi, Y.; Thermal degradation of formamidinium based lead halide perovskites into Sym -triazine and hydrogen cyanide observed by coupled thermogravimetry-mass spectrometry analysis. *J. Mater. Chem. A* 2019, 7(28), 16912-16919. doi:10.1039/c9ta06058h
10. Kroll, M.; Öz, S.D.; Zhang, Z.; Ji, R.; Schramm, T.; Antrack, T.; Vaynzof, Y. ; Olthof, S. ; Leo, K. ; Insights into the evaporation behaviour of FAI: material degradation and consequences for perovskite solar cells. *Sustain. Energy Fuels.* 2022. doi:10.1039/d2se00373b
11. Wei, H.; Chen, S.; Zhao, J.; Yu, Z.; Huang, J.; Is Formamidinium Always More Stable than Methylammonium? *Chemistry of Materials.* 2020, 32(6), 2501-2507. doi:10.1021/acs.chemmater.9b05101
12. Bracesco, A.E.A.; Burgess, C.H.; Todinova, A.; Zardetto, V.; Koushik, D.; Kessels, W.M.M.; Dogan, I.; Weijtens, C.H.L.; Veenstra, S.; Andriessen, R.; Creatore, M.; The chemistry and energetics of the interface between metal halide perovskite and atomic layer deposited metal oxides. *Journal of Vacuum Science & Technology A.* 2020, 38(6). doi:10.1116/6.0000447
